# Optimization and High Level Production of Recombinant Synthetic Streptokinase in *E. coli* Using Response Surface Methodology

**DOI:** 10.22037/ijpr.2019.1100636

**Published:** 2019

**Authors:** Mojtaba Aghaeepoor, Ali Akbarzadeh, Farzad Kobarfard, Ali Akbar Shabani, Ehsan Dehnavi, Sanaz Jamshidi Aval, Mohammad Reza Akbari Eidgahi

**Affiliations:** a *Department and Biotechnology Research Center, Semnan University of Medical Sciences, Semnan, Iran.*; b *Student Research Committee, Semnan University of Medical Sciences, Semnan, Iran. *; c *Gene Transfer Pioneers (GTP) Research Group, Shahid Beheshti University of Medical Sciences, Tehran, Iran.*; d *Department of Medicinal Chemistry, School of Pharmacy, Shahid Beheshti University of Medical Sciences, Tehran, Iran.*

**Keywords:** Streptokinase, Response surface methodology, Expression optimization, Synthetic gene, Escherichia coli

## Abstract

Streptokinase (SK) is an extracellular protein comprising 414 amino acids with considerable clinical importance as a commonly used thrombolytic agent. Due to its wide spread application and clinical importance designing more efficient SK production platforms worth investigation. In this regard, a synthetic SK gene was optimized and cloned in to pET21b plasmid for periplasmic expression. Response surface methodology was used to design a total of 20 experiments for optimization of IPTG concentration, post-induction period, and cell density of induction (OD_600_). The optimum levels of the selected parameters were successfully determined to be 0.28 mM for IPTG concentration, 9.889 H for post induction period, and 3.40768 for cell density (OD_600_). These settings result in 4.14fold increase in SK production rate of optimum expression conditions (7663 IU/mL) in comparison to the primary expression conditions (1853 IU/mL). Achieving higher yields of SK production in shake flask could lead to more cost effective industrial production of this drug which is the ultimate aim of SK production studies.

## Introduction

Streptokinase (SK) (EC 3.4.99.22) is innately secreted by many pathogenic strains hemolytic streptococci as a single chain (47 kDa) extracellular protein, comprising 414 amino acids by a 26 amino acid signal peptide. This protein is of considerable clinical importance as a commonly used thrombolytic agent (1). Reperfusion with thrombolytic agents is one of the possible approaches established to treat acute ischemic stroke. Although symptomatic cerebral bleeding and reperfusion-associated injury are among the risks associated with thrombolytic therapy in patients with acute myocardial infarction, the success achieved with this approach as renewed the interest in reperfusion with thrombolytic agents like SK (2). Despite the fact that originally SK is not a protease, it gives rise to plasmin upon complexation with plasminogen, which in turn dissolves the fibrin network of blood clots and solubilizes degradation products (3, 4). The low cost of producing SK, along with its fewer secondary effects makes it an attractive agent in comparison to tissue plasminogen activator as the major enzyme responsible for clot breakdown. Taken together, it is apparent that designing more efficient SK production platforms is worth investigation.

Small yields of SK production in native hosts, pathogenicity of the native host, and association of other antigenic molecules like Streptodornase are the challenges which lie ahead of implementing high yield production (5). However, heterologous production of SK in *Escherichia coli* (*E. coli*) as the most widely used prokaryotic system for the synthesis of heterologous proteins would circumvent these limitations (6, 7). Rapid growth, cheap cultivation, well studied genetic, and physiological background, various cloning vectors and high expression level in large scale cultures are the main factors which have led to the development of *E. coli* into a highly successful system for the production of various heterologous proteins (8-10). Since heterologous expression of proteins by *E. coli* is dependent on the composition of the culture medium (the expression conditions, expression vector design, promoter strength, expression host strain, and codon usage) the optimization of these factors is highly recommended before industrial scale production (11). Response surface methodology (RSM) is a compelling method to design the minimum number of experiments to find the optimum conditions for heterologous expression.

In this study, the SK gene was designed and optimized for prokaryotic expression and cloned into pET21b expression vector. Thereafter, the RMS approach was employed to optimize the expression conditions. A total number of 20 experiments were carried out to reach the optimum points for three significant cultivation conditions: isopropyl- β-D thiogalactopyranoside (IPTG) concentration, post-induction period and cell density of induction (OD_600_). The optimization process resulted in high preplasmic SK expression, which is of great significance for industrial production. 

## Experimental

*Chemicals, bacterial strains, vectors and DNA techniques*

Bacterial growth was done in Luria and Bertani (LB) medium (Merck, Germany) at 37 °C with shaking at 200 rpm. Restriction enzymes were provided from Takara (Shiga, Japan). Human plasminogen and S-2251 were purchased from Sigma Chemicals Company (USA). All chemicals used in laboratory were analytical grade. *E. coli* DH5a (Stratagene, USA) (f-gyr A96 Nalr, recA1 relA1 Thi-1 hsdR17 r-k m+k) was used as the primary host for the transformation, BL21 (DE3) pLysS (f -ompt hsdB, rB¯ mB¯, dcm gal, DE3, pLYsS cmr) was used to express the SK recombinant protein. The pET-21b (Novagen) was utilized for over-expression of recombinant protein.

*Codon optimization, cloning and construction of the Streptokinase expression vector*

The codon preferences of *E. coli* and *Streptococcus pyogenes (S. pyogenes)* are sig­nificantly different. The DNA coding sequence (1323 bp) of Streptokinase from *S. pyogenes* (GenBank accession no. M19347.1) was taken. Codon optimization was carried out to the codon preference of *E. coli* genes using NCBI-related database at (http://www.kazusa.or.jp/codon). The optimized SK gene with PelB as a signal sequence, flanked by *Nde*I and *Bam*HI restriction sites, was synthesized by Shinegene company (China) (GenBank accession no. KT156726.1) then cloned into pUC57 plasmid called pUC-SK. The synthetic PelB-SK was inserted into the pET-21b between the *Nde*I and *Bam*HI restriction sites. The ligated products were transformed into *E. coli* BL21 (DE3) plysS competent cells by the CaCl_2_ method. Screening was done on LB + 100 mg/mL ampicillin and correctness of cloning was confirmed by colony-PCR and sequencing.

*Induction and Expression of Recombinant Streptokinase in E. coli DE3*

A single colony of *E. coli* BL21 (DE3) harboring recombinant plasmid (pET21b-PelB-SK) was inoculated in 5 ml of LB medium culture at 37 °C with shaking at 200 rpm overnight and supplemented with 100 mg/mL of ampicillin. To induce the expression of SK protein, 0.5 mM (IPTG) was used when the cell density was reached to OD_600 _= 0.8 in the shake flask experiments. The expression was done for 12 h.

*Isolation of peripelasmic SK, renatuartion of inclusion bodies and molecular weight analysis*

The cells in culture were harvested by centrifugation at 4500×g for 10 min at 4 °C and periplasmic expression were obtained by exposing the cell pellet with an equal volume of STE buffer (1 mg lysozyme/mL, 20% (w/v) sucrose, 30 mM Tris/HCl (pH 8.1), 1 mM EDTA) on ice for 10 min. Cell debris was separated by centrifugation according to our previous paper (12).

The separated periplasmic SK was as insoluble inclusion bodies, so it needed a renaturation to reach an active form of recombinant enzyme. First the inclusion pellets were solubilized in 8 M urea buffer at pH 8. The mixture was incubated at 25 °C for 1 h before the insoluble parts were removed by centrifugation. The solution was then diluted with phosphate buffer (pH 10.7) for SK renaturation. The solution was dialysis against the buffer [20 mM Tris/HCl pH 8.0, 50 mM NaCl, 1 mM EDTA] at 4 °C overnight. 

Recombinant SK protein was analyzed by 15% sodium dodecyl sulfate polyacrylamide gel electrophoresis (SDS-PAGE). The protein band of recombinant SK was visualized by Coomassie Blue staining. The amount of total protein was determined by the Bradford method, using bovine serum albumin (BSA) as a standard.

*Overexpression of recombinant Streptokinase using response surface methodology*

Central Composite Design (CCD) and Response Surface Methodology (RSM) were used for optimization of recombinant Streptokinase expression level in *E. coli*. Minitab 16 software (Minitab Inc., USA) was taken to design the experiment and optimization of three significant cultivation conditions: IPTG concentration, post-induction period and cell density of induction (OD_600_). Twenty experiments contain six replicated center points were designed and carried out. The analyses of experimental data were carried out statistically by regression method:

Y = β0 + ∑βi xi + ∑βiixii 2 + ∑βijxi xj + ε

Where Y is the predicted streptokinase mRNA percentage, β0 is a constant coefficient, βi the linear coefficient, βii the quadratic coefficient and βij the cross-product coefficient. Xi and Xj are input independent variable levels, while ε is the residual error. Design Expert software 7.0.0 was employed for Data analysis of experimental design and surface response methodology. The transcriptional level of recombinant Streptokinase was measured by Real Time PCR method in different conditions. Recombinant *E. coli* host cells transformed with pET21b-PelB-SK vector without induction were used as negative control.

*Quantitative analyses by real-time PCR and ΔΔCt method*

Total RNA from *E. coli* BL 21 cells contain recombinant SK were isolated using Trizol reagent (Life Technologies, USA) following the standard protocol. First, the cells were homogenized in 1 mL Trizol solution and 200 μL chloroform was added to samples and mixed completely for 3 min. Then, the mix was centrifuged at 12,000×g for 15 min at 4 °C. The upper aqueous phase was transferred carefully into new tube without disturbing the interphase and equal volume of isopropanol was added in the tube. The mixtures were thoroughly resuspended and incubated in -20 °C for 30 min and centrifuged at 12,000 ×g for 10 min at 4 °C. The precipitated RNA pellets were washed with 1 mL ethanol (75%, v/v). RNA pellets were recovered after centrifugation at 12,000 ×g for 5 min at 4 °C. RNA samples were allowed to air-dry for 2–3 min and then resuspended in 30 mL diethyl pyrocarbonate-treated water (Life Technologies, USA). The extracted RNA was quantified by measuring absorbance at 260/280 nm by Nanodrop and the quality of RNA purified was checked by gel electrophoresis. cDNA synthesis was carried out with Thermo Scientific cDNA synthesis kit. Reverse transcription was followed using 50 mg total RNA (maximally in 20 mL) and 1 mL random hexamer primers. The volume of the assay mixture was adjusted to 12 mL with RNase-free water, and then the mixture was incubated for 5 min at 70 °C, followed by incubation for 10 min at room temperature to allow the primers to anneal with the RNA.

**Figure 1:**
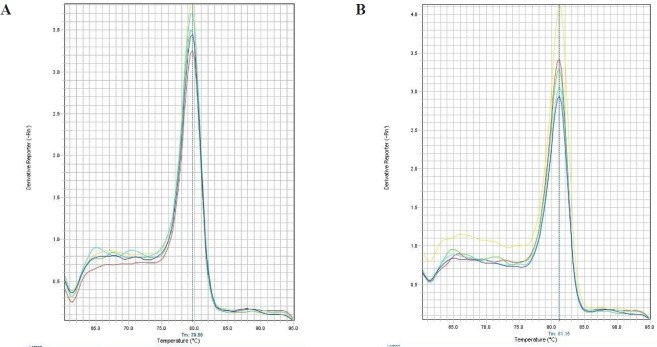
Melt curve for Real Time PCR experiments. (A) Melt curve for 16srRNA gene as an internal control and (B) Melt curve for Streptokinase gene

For analysis of streptokinase expression level, Real Time PCR was performed by using Power SYBR® Green PCR Master Mix (life technology), according to manufacturer’s instructions (Applied Biosystems, USA). The kit has a Hot-start *Taq* DNA polymerase. All samples were analyzed in duplicate and the average value is reported. For determination of the mRNA level, 16S rRNA was used as internal control gene. The primers used for this study were designed using Web-based Oligo7 Primer Analysis Software. The primers for streptokinase were: sense 5- CATAAACTGGAAAAAGCCGATCTG -3 and antisense 5- GACCGCTCAGCAGAAATTCTTG -3. The primers for 16S were: sense 5- CTACGGGAGGCAGCAGTGG -3 and antisense 5-TATTACCGCGGCTGCTGGC -3. The StepOne™ Real-Time PCR Systems (ABI) was used to detection relative quantification. The amplification reactions were done under following conditions: 10 min at 95 °C, followed by 45 cycles at 95 °C for 15 sec, 60 °C for 1 min. Melting curve program was set to 60-95 °C with a heating rate of 0.1 °C per second and a continuous fluorescence measurement. In order to identify the specificity of amplification products a dissociation curve was plotted (Figure 1). The 2^-ΔΔCt^ method was used to analyze the relative changes in the level of gene expression (13).

*Determination of Streptokinase activity*

SK activity was assayed using by chromogenic substrate method that was an endpoint method. The Streptokinase transformed plasminogen to plasmin in solution, in the existence of chromogenic substrate S-2251 (H-D-valyl-L-leucyl-L-lysine-p-nitroanilide dihydrochloride; (Sigma, USA), in the absence of fibrin.

Substrate solution included a mixture of 1 mL of 0.5 M Tris–HCl pH 7.4, 1 mL of 3 mM S- 2251 and 5 μL of 10% Tween 20. This solution was kept at 37 °C and immediately before use, 45 μL of human-plasminogen solution (1 mg/mL) was also added to substrate solution. Streptokinase solution tested at different concentrations for the dose-response curve. Streptokinase diluted in 10 mM of Tris–HCl (pH 7.4) at 37 °C, 0.1 mM NaCl and 1 mg/mL albumin to reach 4.0, 2.0, 1.0, 0.5 IU/mL concentrations and maintained at 37 °C in a microtiter plate. In the test samples, the reaction was performed after addition of 60 μL of Streptokinase solution to 40 μL of substrate solution. The absorbance of the wells was measured at 405 nm for 20 min before an endpoint OD was taken immediately. 

## Results

*Gene optimization, cloning and expression of optimized SK gene in E. coli DE3*

Different cells use the same codons with different codon preferences. The coding sequence of SK gene from *S. pyogenes* (GenBank Acc. No. M19347.1) was synthesized with codon optimi­zation of *E. coli*. The optimized synthetic gene (GenBank KT156726.1) and wild-type sequence (GenBank M19347.1) share 78% identity. During SK gene optimization, 275 nucleotides were changed, which led to 235 amino acid codon optimization (56.5%) and deletion of 23 rare codons (Table 1). The GC content increased from 40% to 45%, closer to the average GC content of other highly expressed genes in *E. coli* Kazusa. Moreover, no cryptic splicing sites, internal chi sites and ribosomal binding sites, negative CpG islands, repeat sequences, restriction sites that may interfere with cloning, RNA instability motif (ARE), and mRNA secondary structure were detected to be optimized.

**Figure 2:**
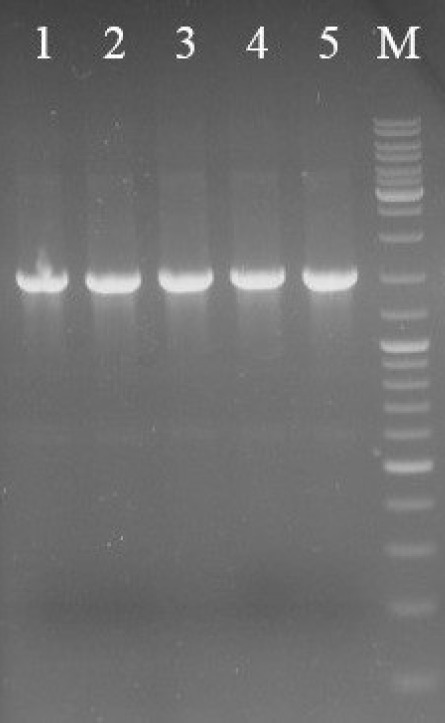
Colony-PCR analysis of transformants indicated that the synthetic SK gene was successfully cloned. Lanes 1-4 are colony-PCR on transformed colonies of *E. coli*, Lane 5 is pUC-SK plasmid as a positive control. Lane M is DNA marker

The SK gene fragment was digested out of the pUC-SK cloning vector and ligated into the pET21b expression vector. The SK gene fragment (~1300 bp) containing streptokinase gene and PelB signal sequence was inserted into *Nde*I/*Bam*HI sites downstream of the T7 promoter region. The *E. coli* BL21 DE3 was transformed by recombinant pET21b-SK plasmid. As shown in Figure 2, screening of transformants by colony-PCR technique indicated that the SK gene was successfully cloned. The accuracy of the cloning was confirmed by sequencing results.

**Figure 3:**
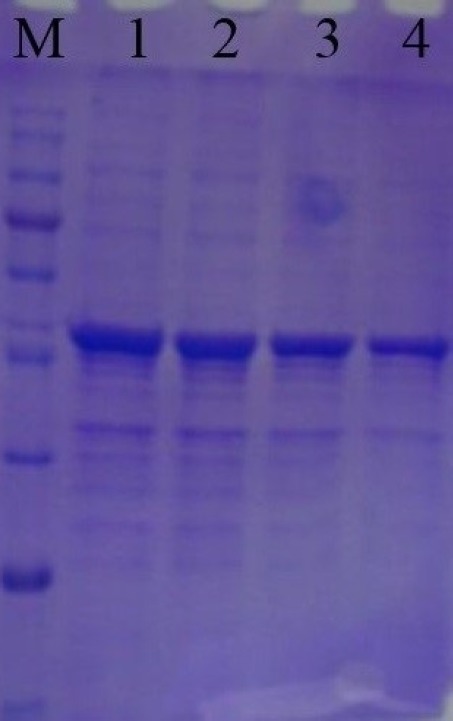
SDS-PAGE analysis of recombinant Streptokinase. Lane M is the protein molecular marker. Lane 1-4 are samples of SK expression at different hours with 47 kDa recombinant protein. Lane 1, 2, 3 and 4 are expression after 8, 6, 4 and 2 h, respectively

Recombinant SK gene was expressed using IPTG as the inducer. The expressed protein was then analyzed by SDS-PAGE method. SDS-PAGE analysis of total lysate of induced *E. coli* BL21 (DE3) demonstrated a protein band in the desired range, with a MW of ~47 kDa (Figure 3). The SDS-PAGE results indicated that the SK was successfully and highly expressed under the induction of IPTG. For quantification, total RNA from *E. coli* BL 21 cells containing recombinant SK RNA was isolated, cDNA was synthesized and the amount of expression was measured by Real Time PCR.

*Experimental design and modeling for SK optimization by RSM*

Following the confirmation of SK expression under the basal conditions, the optimum levels of significant cultivation conditions including IPTG concentration, post-induction period, and cell density of induction (OD_600_) should be achieved. In this regard, the experimental range of each three variables was produced in five levels (-α, -1, 0, +1, +α) (Table 2). Thereafter, 20 experiments including six replications of the central points were designed to optimize the selected parameters (Table 3). Moreover, this table provides the assessed (by real time method) percentage of expressed SK mRNA under the *Actual* column, while the predicted amounts are represented under the *Predicted* column. The provided predicted levels of SK activity (on mRNA expression level) as the function of IPTG Concentration (A), Post Induction Period (B) and Cell Density (C) were calculated based on the equation obtained by regression method: 

Y (SK expressed mRNA Percentage) = 89.21 – (1.26 A) + (7.73 B) + (5.93 C) – (2.26 A^2^) – (3.91 B^2^) – (9.65 C^2^) + (1.20 AB) + (1.75 AC) – (1.75 BC)

**Figure 4:**
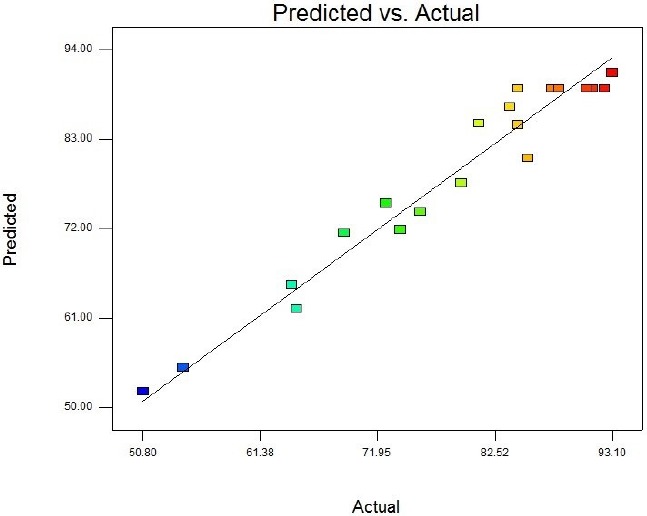
Predicted response versus actual value

**Figure 5.**
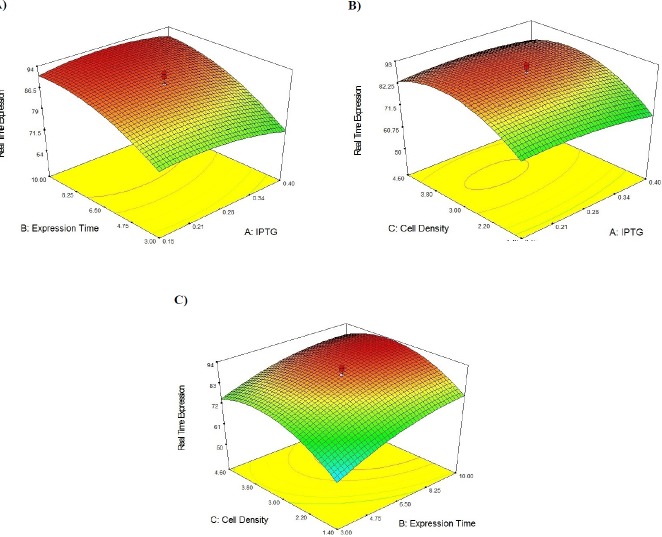
3D response surface for Streptokinase production by *E. coli*. The effect of two variables while the other two are held at 0 levels. (A) It shows the effect of IPTG concentration and expression time on secreted SK activity, (B) It shows the effect of IPTG concentration and cell density on secreted SK activity, (C) It shows the effect of expression time and cell density on secreted SK activity

**Figure 6:**
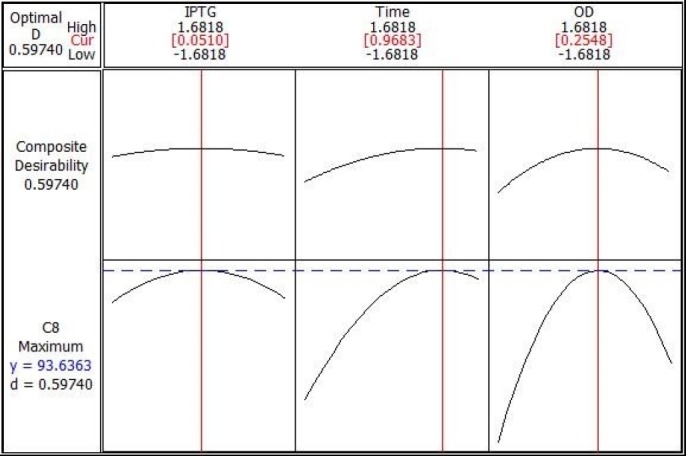
Optimization plot for highest recombinant SK activity

**Table 1:** Codon optimization

**Codon**	***E. coli*** **Kazusa (%)**	**Wild STK (%)**	**Syn. STK (%)**	**Codon**	***E. coli *** **Kazusa (%)**	**Wild STK (%)**	**Syn. STK (%)**
**Ala (A)**				**Asn (N)**			
GCA	21	24	68	AAT	45	29	67
GCT	16	64	0	AAC	55	71	33
GCG	36	0	8	**Pro (P)**			
GCC	27	12	24	CCA	19	28	0
**Cys (C)**				CCT	16	39	11
TGT	45	0	0	CCG	52	22	89
TGC	55	0	0	CCC	12	11	0
**Asp (D)**				**Gln (Q)**			
GAT	63	57	92	CAA	35	93	36
GAC	37	43	8	CAG	65	7	64
**Glu (E)**				**Arg (R)**			
GAA	69	68	79	AGA	4	11	0
GAG	31	32	21	AGG	2	0	0
**Phe (F)**				CGA	6	22	0
TTT	57	81	75	CGT	39	44	67
TTC	43	19	25	CGG	10	6	0
**Gly (G)**				CGC	39	17	33
GGA	11	26	0	**Ser (S)**			
GGT	34	22	68	AGT	15	14	7
GGG	15	26	0	AGC	28	32	89
GGC	40	26	32	TCA	12	25	4
**His (H)**				TCT	15	18	0
CAT	57	67	92	TCG	15	4	0
CAC	43	33	8	TCC	15	7	0
**Ile (I)**				**Thr (T)**			
ATA	7	9	0	ACA	13	37	3
ATT	51	59	64	ACT	17	20	0
ATC	42	32	36	ACG	27	10	0
**Lys (K)**				ACC	44	33	97
AAA	77	79	100	**Val (V)**			
AAG	23	21	0	GTA	15	4	0
**Leu (L)**				GTT	26	48	68
TTA	13	34	0	GTG	37	8	32
TTG	13	18	0	GTC	22	40	0
CTA	4	16	0	**Tyr (Y)**			
CTT	10	13	0	TAT	57	57	91
CTG	50	11	100	TAC	43	43	8
CTC	10	8	0	**Stop (TERM)**			
**Try (W)**				TAA	64	100	100
TGG	100	100	100	TAG	7	0	0
**Met (M)**				TGA	29	0	0
ATG	100	100	100	GC%	51	40	45

Codons with low frequency (10%) are highlighted in blue (light) and the most preferred codon for each amino acid is highlighted in gray (dark). The native Streptokinase gene has lower preferred codons with high frequency than optimized gene. For gene optimization, rare codons frequency (56.49%) were omitted completely. The GC content increased from 40% to 45%, closer to the average GC content of other highly expressed genes in *E. coli *Kazusa.

**Table 2:** Experimental range of variables and coded values of three variables used in Central Composite Design

**Variable Levels**	**Component**	**Level**				
		**-α**	**-1**	**0**	**+1**	**+α**
A	IPTG Concentration (mM)	0.065	0.15	0.275	0.4	0.485
B	Post Induction Period (h)	0.62	3	6.5	10	12.38
C	Cell Density (OD600)	0.312	1.4	3	4.6	5.688

**Table 3:** CCD with measured and predicted response with transcription level of STK as a response

**Run No.**	**Factor A**	**Factor B**	**Factor C**	**Variations of streptokinase mRNA (%)**	
	**IPTG Concentration**	**Post Induction Period**	**Cell Density**	**Actual**	**Predicted**
1	-1	-1	-1	64.6	62.2
2	1	-1	-1	54.4	53.8
3	-1	1	-1	79.5	78.8
4	1	1	-1	72.7	75.1
5	-1	-1	1	75.8	74.1
6	1	-1	1	68.9	72.6
7	-1	1	1	84.6	83.6
8	1	1	1	83.9	87.0
9	-1.68	0	0	81.1	84.9
10	1.68	0	0	85.5	80.7
11	0	-1.68	0	64.2	65.2
12	0	1.68	0	93.1	91.2
13	0	0	-1.68	50.8	51.9
14	0	0	1.68	74	71.9
15	0	0	0	88.3	89.2
16	0	0	0	84.6	89.2
17	0	0	0	91.3	89.2
18	0	0	0	90.8	89.2
19	0	0	0	87.7	89.2
20	0	0	0	92.4	89.2

**Table 4:** Analysis of variance (ANOVA) for response surface quadratic model for the Streptokinase production

**Source**	**Sum of Square**	**F-value**	**Prob > F**
Model	315.22	25.31	0.0001
A-IPTG mM	21.66	1.74	0.2166
B-Expression Time (h)	816.6	65.56	0.0001
C-Cell Density (OD600)	480.63	38.59	0.0001
AB	11.52	0.92	0.3589
AC	11.05	0.89	0.3686
BC	11.04	0.89	0.3686
A2	73.71	5.92	0.0353
B2	219.82	17.65	0.0018
C2	1342.23	107.76	0.0001

*Validation of the model and experimental confirmation*

Table 4 contains the data pertaining to the confirmation of statistical significance of the above equation by an F-test and the analysis of variance (ANOVA) for response surface quadratic model. The Model F-value of 25.31 with a very low probability value [(Prob > F) < 0.0001] confirms that the model is significant and there was only a 0.01% chance that the Model F-Value could occur as a consequence of the noise. Values of "Prob > F" less than 0.0500 indicate model terms are significant. In this case B, C, A2, B2, and C2 are significant model terms. Values greater than 0.1000 indicate the model terms are not significant. If there are many insignificant model terms (not counting those required to support hierarchy), model reduction may improve the model. Moreover, the diagnostic plots were used for estimating the adequacy of the regression model. The R^2^ coefficient was determined to check the fitting of the model. The closer R^2^ values to 1 show the stronger model and the better prediction of response. The actual values are the result obtained for a specific run and the predicted values are obtained from the independent variables in the CCD model. The R^2^ value was calculated to be 0.96, therefore our results reveals that the regression model for SK overexpression fits to the experimental values (Figure 4). The effect of each factor on the SK activity (on the mRNA expression level) and their optimum amount are depicted by 3D surface plot (Figure 5). The optimum levels of the selected parameters were determined to be 0.28 mM for IPTG Concentration, 9.889 H for post induction period, and 3.40768 for cell density (OD_600_). Predicted maximum SK activity (on the mRNA expression level) was 93.64 % which is in concordance to the 94.27% SK activity (on the mRNA expression level) obtained during the experiments (Figure 6).

*Refolding and comparison of SK activity before and after optimization*

Ultimately, the total activity assay of the SK cultured in the optimum amounts of IPTG concentration, post induction period, and cell density indicates a significant increase compared to the SK activity under the primary expression conditions (0.5 mM for IPTG Concentration, 12 H for post induction period and 0.8 for cell density (OD_600_)). The achieved activity results indicate a 4.14fold increase in SK activity of optimum expression conditions (7663 IU/mL) in comparison to the primary expression conditions (1853 IU/mL).

## Discussion

Achieving an optimum expression condition for a recombinant protein could be affected by various factors (14, 15). It should be taken into consideration that the expression of a plasmid in the host cell exerts a metabolic burden which could end with reduced specific growth rate and biomass content and plasmid instability (16). Moreover, the onset of glucose overflow metabolism and acetate formation as two detrimental factors for recombinant protein production determines the upper limit of the specific growth rate (17-19). These parameters accentuate the necessity of obtaining an optimum condition for overexpression of recombinant proteins. In this regard, we have employed the RSM, which is proved to be one of the most accurate multivariate analysis methods, to determine the optimum culture conditions for SK expression by simultaneously changing of IPTG concentration, post-induction period, and cell density of induction (OD_600_).

Different bacterial species do not share the same codon preference for their translation processes. Thus, biased codon usage is one of the major factors affecting the heterologous gene expression and should be dealt with properly. Decreased mRNA stability and transla­tion rate are the consequences of existing rare codons and high G+C contents could lead to reduced translational yields or even failed expression (20). Codon optimization as a genetic technique in which the existing rare codons of a species are replaced with a set of more favorable host codons throughout the whole gene could enhance the recombinant protein expression by 2-3 folds (20-23). Moreover, employing gene optimization algorisms could benefit simultaneous optimization of cryptic splicing sites, internal chi sites, and ribosomal binding sites, negative CpG islands, repeat sequences, restriction sites that may interfere with cloning, RNA instability motif (ARE) and mRNA secondary structure, the latter of which affects the translation efficiency (24). This technique was employed to resolve the varying codon usage preference between the *S. pyogenes* and *E. coli* to achieve optimum expression of the SK gene in a host’s cellular system. Chemical synthesis of the target gene is rationally the most appropriate way to get the codon-optimized gene. Introduction of the pelB signal peptide to the SK gene through a synthesis process would guide the protein into periplasmic space. Periplasmic expression could additionally provide the SK with separation from other impurities in cytoplasmic space, an oxidizing medium for the formation of disulfide bonds, and keeping its activity and biological structure (25). 

It has been reported that IPTG concentration, post induction period and cell density are among the most important production conditions to be optimized for high yield recombinant protein expression (26). The conventional approach to optimizing production conditions is to vary one parameter at a time whilst keeping the others constant. However, due to the large number of required experiments this approach is not practical when numerous parameters are taken into account. Aside from the cumbersome nature of this approach, it could lead to misinterpretation of results when the interaction between different variables is present (27). RSM is a commonly used alternative method to overcome the aforementioned snags. It is a mathematic and statistical tool used to design optimization experiments, to build models, and to study the interactions within various bioprocess parameters, whilst also running the smallest number of experiments possible (28). Using 5 levels of each variable in the experimental design seems to be more efficient than using 3 levels to arrive at the best results. In addition to determining the optimum conditions, the optimization in 5 levels reveal the accuracy of the selected range of variables. The point prediction tool of the software was used to determine the optimum value of IPTG concentration (0.28 mM), post induction period (9.889 H), and cell density (3.40768). Furthermore, to have a better grasp on the three factors of optimal SK production, the models were presented as 3-D response surfaces. The obtained results revealed that our RSM approach successfully optimized SK production up to a 4.14fold increase. To the best of our knowledge, the obtained yield of periplasmic SK production within the shake flasks is the highest yield reported so far (29-32).

Although the *E. coli *expression system is a highly characterized one and various expression settings have been developed based on this host, the beginning of the log phase of the bacterial growth is thought to be the best stage for protein expression. However, our observations contradict this, as we have demonstrated that the highest amount of recombinant SK production was reached at midway, and close to the end of the log phase, which occurred after induction. Studies published by Galloway *et al.* Chae *et al.* and Samarin *et al.* have reported high yield recombinant protein expression close to the end point of the log phase (33-35). They have reported escalation of soluble expression of the target protein and their diminished proteolysis in the cytoplasm. These studies, along with the study conducted by J.Ou *et al.* were in concordance with our results (36). It should be noted that this finding is applicable to the heat shock induced promoters as well as Lac based promoters (36). It has generally been suggested that the *E. coli *cells at their midway of log phase or close to the end of log phase behave like the cells at the beginning of their log phase (with high physiological activity) regarding the transcription, translation, and protein folding processes. Moreover, the secretion of the expressed recombinant protein seems to be alleviated close to the end of the log phase. These phenomena could be rooted in the fact that the metabolic flow of the bacteria is diverted towards producing the target protein. This could be the rationale behind the growth halt observed an hour after induction at the end of this phase. The concentration of the employed IPTG is the other factor that should be considered for optimum protein expression. The high cost and potential cytotoxicity of IPTG makes it an imperative optimization target. IPTG needs further development due to its ability for significant reduction in growth rate and production of bacterial proteases degrading heterologous proteins at high concentrations. Although employed, IPTG concentration for induction ranges widely from 0.005 to 5 mM, our study finds out the optimum amount of IPTG to be as low as 0.28 mM (37). In this regard, Larentis *et al.* have already reported that employing IPTG at a concentration 10 fold lower than usually used could result in optimum expression of recombinant (38).

In conclusion, the employed RSM successfully determined the optimized conditions for recombinant SK production. Achieving higher yields of SK production in shake flasks could lead to more cost effective industrial production of this drug which is the ultimate aim of SK production studies. Although our investigation have reached a 4.14fold increase in the production rate, it seems that optimization of other influential parameters of protein expression would bring about higher yields of SK production. 
